# Applause to *PLoS Medicine* for Initiating Student Forum

**DOI:** 10.1371/journal.pmed.0020223

**Published:** 2005-07-26

**Authors:** C. Jairaj Kumar, Abhizith Deoker

**Affiliations:** **1**Kasturba Medical CollegeMangaloreIndia

We congratulate and acknowledge *PLoS Medicine* for initiating a students' forum [1] and for its efforts to encourage students' participation across the globe. There is really a need for medical students, especially from the developing world, to be actively conducting research, reading journals, publishing papers, and staying in touch with current developments in the field of medicine. Many developing countries lack a national-level student medical journal for students to exchange their views and ideas, which thereby pacifies their thinking and makes them hypnotic to issues such as the influence of drug companies and the neglected health problems of poorer countries.

It will be really motivating for students from developing countries to actively take part in debate through the Student Forum of *PLoS Medicine*, which is composed of articles selected by student representatives across the world. The unique integration of student associations with *PLoS Medicine*, and the journal's policy of not publishing advertisements for drugs or medical devices, will also enlighten students about the influence of drug companies in medical practice and enable students to realize their priorities in poorer countries for the future. Thereby, students may focus their attention on becoming professionals in developing new strategies to combat killer infectious diseases like malaria and tuberculosis, and malnutrition—such as vitamin deficiencies among children and iron deficiency among pregnant women—that are dreaded and very common in poorer countries.
